# Celastrol Alleviates Airway Hyperresponsiveness and Inhibits Th17 Responses in Obese Asthmatic Mice

**DOI:** 10.3389/fphar.2018.00049

**Published:** 2018-01-31

**Authors:** Zeyu Zeng, Xixi Lin, Rongying Zheng, Hui Zhang, Weixi Zhang

**Affiliations:** ^1^Department of Pediatric Allergy and Immunology, The Second Affiliated Hospital and Yuying Children’s Hospital of Wenzhou Medical University, Wenzhou, China; ^2^Department of Pharmacy, The Second Affiliated Hospital and Yuying Children’s Hospital of Wenzhou Medical University, Wenzhou, China

**Keywords:** celastrol, obese, asthma, airway hyperresponsiveness, T help cell 17

## Abstract

Severe airway hyperresponsiveness (AHR) is a clinical feature of asthma, which has been associated with obesity and has shown a poor response to standard asthma treatments such as glucocorticoids. Numerous studies have shown that Interleukin (IL)-17 producing CD4^+^T cells (Th17 cells), which could be inhibited by celastrol, is essential in mediating steroid-resistant AHR. The following study investigates the impact of celastrol and its mechanism on the regulation of AHR in murine model of obesity and asthma. C57BL/6 mice were sensitized by intraperitoneal injection of ovalbumin (OVA) on day 1 and 13 starting from 12th week, which was followed by aerosol OVA challenge that lasted for 30 min per daily for 7 consecutive days starting from 16th week. Diet-induced obesity (DIO) mice were fed a high fat diet (HFD) for 16 weeks. Celastrol was administrated orally for 7 consecutive days, 30 min before every challenge in DIO-OVA-induced mice. Lung functions were analyzed by measuring the airway resistance (Rn) and methacholine (MCh) AHR, while H&E staining was used to examine histological changes in the lungs. Immunohistochemistry was used to observe IL-17A protein in lung tissues; flow cytometry to detect the proportion of Th17 cells in CD4^+^T cells. The concentration of cytokines IL-17A in serum was assessed by standardized sandwich ELISA, while the expression of IL-17A mRNA in lung was examined by quantitative real-time RT-PCR. Briefly, our data indicated that celastrol reduced body mass in DIO-OVA-induced obesity and asthma. Both baseline Rn and MCh AHR were significantly lower in celastrol group. Moreover, celastrol treatment decreased the frequency of Th17 cell expansion and reduced the production of IL-17A in both lung and serum. To sum up, our findings indicated that Th17 and its cytokine measured in the spleen and lung were closely associated with AHR. In addition, celastrol has shown the ability to suppress AHR through Th17 inhibition in obese asthmatic mice.

## Introduction

Asthma is a common chronic inflammatory disease of the airways. Airway hyperresponsiveness (AHR) is one of the common clinical features of asthma. Even though asthma is generally well regulated using conventional therapies, e.g., using inhaled corticosteroids; there are still several phenotypes that are insensitive to steroids. Obesity has been suggested to have a substantial role in the development and control of asthma (Permaul et al., 2014). For example, it decreases the efficacy of asthma-control medications, making asthma very difficult to treat. As the relationship between obesity and asthma grew closer, Children’s Asthma International Consensus (ICON) have classified the asthma in obese individuals as a special “obese-asthma” phenotype (Papadopoulos et al., 2012). Obese asthma is regarded as a steroid-resistant asthma (Wenzel, 2012). According to existing research, AHR is characteristic feature of both asthma and obesity. It has been shown that increased body mass leads to the occurrence of AHR (Arteaga-Solis et al., 2013). Nevertheless, obese asthma is associated with more severe AHR (Kim et al., 2015). Even though previous studies have proved that AHR is a common pathogenesis between obesity and asthma, the exact mechanisms underlying AHR in obese asthma remain unclear and need to be further investigated.

Researches have shown that interleukin (IL)-17 producing CD4^+^T cells (Th17 cells) is essential in mediating steroid-resistant AHR in mice (McKinley et al., 2008). The importance of Th17 cells lies in the ability of IL-17 to induce neutrophil chemotaxis and antiapoptotic properties to mediate steroid-resistant asthma (Saffar et al., 2011; Ano et al., 2013; Morishima et al., 2013). Barczyk et al. (2003) have suggested that the levels of IL-17 in sputum are correlated with AHR to methacholine in subjects with asthma. Moreover, our previous studies have shown that Th17 cells are involved in the pathogenesis of asthma, which suggests that airway inflammation could be alleviated by inhibiting Th17 differentiation, and decreasing IL-17 production in ovalbumin (OVA)- induced mice (Zhang et al., 2015). On the other hand, Th17 cells are also involved in the obesity. A high-fat diet augments Th17 cell development in both mice and humans (Endo et al., 2015). Mice fed with high-fat diets have shown to develop AHR, which in turn is correlated with Th17 cells and IL-17A levels (Mathews et al., 2014). Accordingly, since Th17 is involved in the regulation of AHR in both obesity and asthma, we speculate that Th17 might be involved in mediating AHR in obese asthma. This would make it a critical therapeutic target, which if suppressed might alleviate AHR in obese asthmatics.

Celastrol is an effective natural bioactive compound extracted from the roots of *Tripterygium wilfordii.* It has proved to be very valuable in anti-inflammatory, anti-cancer, anti-rheumatic and autoimmune diseases (Kannaiyan et al., 2011). It has also proved to have an amazing effect on body mass reduction in mice (Liu et al., 2015). Interestingly, celastrol has also been found to suppress airway inflammation and AHR in allergic asthma (Kim D.Y. et al., 2009). Moreover, celastrol could be used as a potential adjunct/alternative for rheumatoid arthritis (RA) therapy due to its ability to reduce Th17 cells in the rat adjuvant-induced arthritis (AA) model (Cascão et al., 2012; Astry et al., 2015). Nevertheless, the effect of celastrol on AHR in obese asthmatics has still not been clarified. Therefore, we observed the regulation of AHR using celastrol on a model of obesity and asthma, and identified its underlying mechanisms.

## Materials and Methods

### Animals

Male C57BL/6 mice (3–4 weeks old; weighing 15–20 g) were purchased from Shanghai SLAC Laboratory Animal Center (Shanghai, China) and were housed in an environment with temperature of 22 ± 1°C, relative humidity of 50 ± 1% and a light/dark cycle of 12/12 h. All animal studies (including the mice euthanasia procedure) were done in compliance with the regulations and guidelines of Wenzhou Medical University institutional animal care and conducted according to the AAALAC and the IACUC guidelines.

**FIGURE 1:**
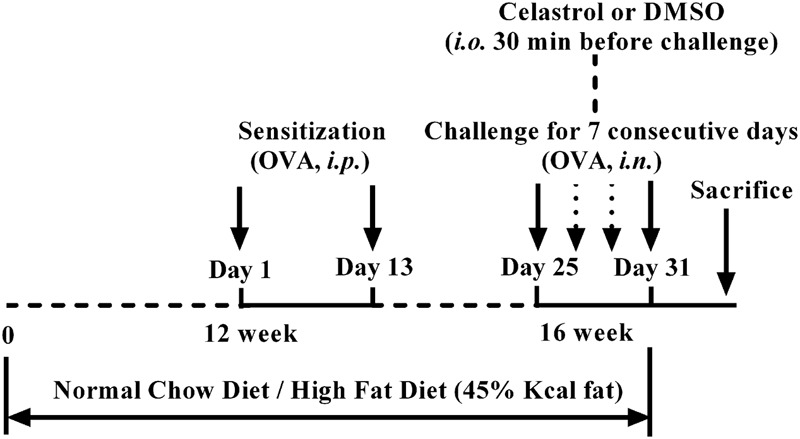
Study design.

Experimental animals were randomly divided in five groups: sham group, OVA+DMSO (vehicle) group, DIO+DMSO group, DIO+OVA+DMSO group and DIO+OVA+celastrol group. An ovalbumin (OVA) – induced asthma model was established according to previously described approach (Chong et al., 2014; Zhang et al., 2013), while the diet-induced obesity (DIO) model was established by feeding mice with high fat diet (HFD) for 16 weeks. The HFD (MD12032, Medicience Ltd, China) contained 45% kcal from fat; the lean mice (used as a control) were fed a normal chow diet (MD12031, Medicience Ltd, China) containing 10% kcal from fat (Wu J. et al., 2013; Wu T. et al., 2013). In order to established an asthma model in lean or DIO mice, mice were sensitized by i.p. injection of 10 μg OVA (Sigma, United States) emulsified in 20 mg Al (OH)_3_ gel in 0.1 mL normal saline (NS) on days 1 and 13 starting from 12th week. They were then challenged with OVA (1 mg/mL) aerosol for 30 min daily for 7 consecutive days starting from 16th week by Jet nebulizer (Pari IS-2 Jet nebulizer, PARI Respiratory Equipment). Sham mice were sensitized and challenged with NS. Schedule of study design was shown in **Figure 1**.

### Treatment with Celastrol

Celastrol (Boyun Biotech Co., Ltd., Shanghai) was dissolved in DMSO (Sigma, USA) as a 20 mg/mL stock solution. Celastrol (10 mg/kg/200 μl) was administered orally for 7 consecutive days, 30 min before every challenge. The volume of DMSO contained in each concentration of celastrol was 20 μl. The control mice were orally administrated with DMSO instead of celastrol 30 min before every challenge, after adjusting to 200 μl with PBS.

### Lung Function Analysis

Lung functions were measured within 24 h following the last allergen challenge. Mice were anesthetized with 1% pentobarbital sodium (50 mg/kg) using i.p. injection. When spontaneous breathing stopped, the trachea was cannulated with a 12G tubing adaptor, and then connected to the forced oscillation technique (FlexiVent, SCIREQ, Canada). The mice were ventilated at 150 breaths/min with a tidal volume (VT) of 0.2 ml and a positive end-expiratory pressure (PEEP) of 2 cmH_2_O using a mouse ventilator (FlexiVent, SCIREQ, Canada). Baseline airway resistance (Rn) was measured at the stage of aerosolized saline nebulizing. Consequently, the mice were challenged with an increasing dose of methacholine (MCh) (3.125 mg/mL, 6.25 mg/mL, 12.5 mg/mL, 25 mg/mL, 50 mg/mL), and Rn was measured at every dose to calculate the percentage of each concentration to baseline.

### Histopathological Examination

After measuring lung function, the middle part of left lung tissue was first fixed with 4% paraformaldehyde for 4 h. It was then dehydrated in ethyl alcohol, embedded with paraffin, cut into 4 μm sections, and stained with hematoxylin and eosin (H&E) staining. Lung tissue slices were evaluated using light microscope (Nikon, Japan) to observe infiltration of inflammatory cells and airway morphology. The degree of allergic airway inflammation was scored based on the following histologic grading system (scored 0–4): absence of peribronchial inflammatory cells; a few scattered peribronchial inflammatory cells involving <25% of the circumference of the bronchus; focal peribronchial inflammatory cells infiltration not completely surrounding a bronchus (i.e., involving approximately 25–75% of the circumference of the bronchus); one definite layer of peribronchial inflammatory cells completely surrounding a bronchus; 2 or more layers of peribronchial inflammatory cells completely surrounding abronchus. In each lung section the mean peribronchial inflammatory score was determined by the sum of scores from all individual bronchioles in the section divided by the number of bronchioles.

### Immunohistochemistry

Lung tissues were immunohistochemically analyzed with IL-17A antibody (Santa Cruz Biotechnology, United States). The sections were counterstained using hematoxylin (Thermo Shandon, PA, United States). The expression of IL-17A was measured using Image-Pro Plus 6.0 via optical density analysis.

### Preparation of Splenic Single-Cell Suspension

The spleen tissue was fragmented into small pieces that were then pressed against nylon mesh with a plunger of a disposable syringe. Erythrocytes were lysed by red blood cell lysis buffer. Cells were washed in fresh PBS.

### Isolation of CD4^+^T Cells

CD4^+^T cells from splenic single-cell suspension were isolated by magnetic cell sorting by positive selection method using mouse CD4^+^T cell isolation kit (MACS, Miltenyi Biotec, Germany) according to the manufacturers’ instruction.

### Flow Cytometry Analysis

FITC-labeled anti-mouse CD4 and PE-labeled anti-mouse IL-17A were used to detect Th17 cells. Matching IgG was used as isotype control. All antibodies were purchased from BD Bioscience, United States. For Th17 cell analysis, CD4^+^T cells (1 × 10^6^/mL) from the spleen tissue were stimulated for 4.5 h with phorbol myristate acetate (PMA) at 100 ng/mL and Ionomycin at 1 μg/mL in the presence of 1.6 μg/mL Monensin (all from Beyotime, China). Cells were collected, washed, and surface-stained with FITC-labeled anti-CD4 antibody at 4C for 20 min in the dark, and then resuspended in Fix/Perm solution according to the manufacturer’s instruction (Invitrogen, United States). They were then stained intracellularly with PE-labeled anti-IL-17 antibody. After washing, cells were resuspended in fixation solution and subjected to FC500 flow cytometer (Beckman Coulter, United States) analysis. Background fluorescence was assessed by the corresponding isotype control antibodies. Data were analyzed with KALUZA software.

### ELISA

The concentration of cytokine IL-17A in serum were assessed by standardized sandwich ELISA according to the manufacturer’s protocol. ELISA kit was acquired from Boyun, Shanghai, China.

### Quantitative Real-Time RT-PCR Analysis

RNA was extracted from lung tissue using Trizol (Invitrogen, United States) according to the manufacturer’s instruction. cDNA was synthesized by reverse transcription with oligo (dT) from total RNA. The quantitative real-time RT-PCR was performed using lightcycler 480 System (Roche, Switzerland) with SYBR Green (Roche, Switzerland). GAPDH was used as internal control. Primers used were as follows: GAPDH, 5′-AAGAAGGTGGTGAAGCAGG-3′ (forward) and 5′-GAAGGTGGAAGAGTGGGAGT-3′ (reverse). IL-17A, 5 ′-TCATGTGGTGGTCCAGCTTTC-3′ (forward) and 5′-CTCAGACTACCTCAACCGTTCC -3′ (reverse). Delta-Delta Ct method was used to express the fold induction of target mRNA after GAPDH normalization.

### Statistical Analysis

Differences between groups were performed using one-way ANOVA (The *post hoc* test utilized following ANOVA was listed in Supplementary Data). The correlation coefficient *r* was generated using Spearman’s rank correlation. *P* < 0.05 was considered statistically significant. All experiments were run in triplicate.

## Results

### Repetitive Oral Delivery of DMSO Did Not Cause Non-specific Inflammation

To exclude the impact of DMSO on the interpretation of the celastrol effect, we examined baseline Rn, MCh AHR, lung expression of IL-17A mRNA and serum IL-17A among different groups. As shown in Supplementary Figure 1, there were no significant differences between DMSO-treated and non DMSO-treated groups.

### Celastrol Reduces the Body Mass of Obese Asthmatic Mice

**FIGURE 2:**
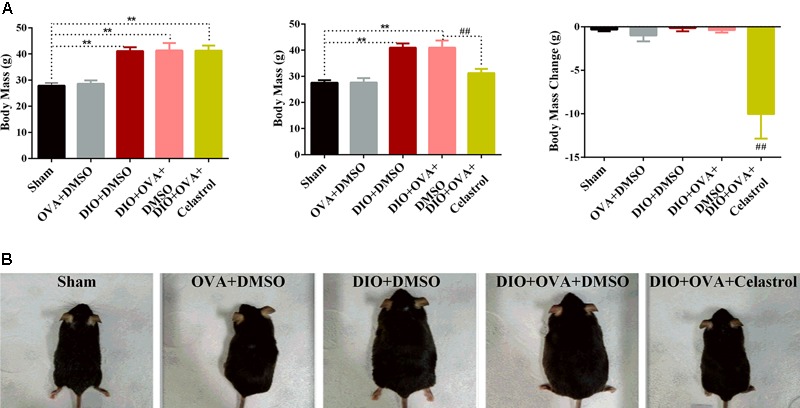
Celastrol reduces body mass of obese asthmatics mice. **(A)** C57BL/6 mice were fed a normal or high fat diet (HFD) for 16 weeks and treated with OVA. Body mass was measured before and after celastrol administration during 7 consecutive days. **(B)** Appearance changes after 7 days of celastrol adminstration. The data were expressed as mean ± SD (*n* = 6). ^∗∗^*P* < 0.01 compared with Sham group, ^##^*P* < 0.01 compared with DIO+OVA+DMSO group.

Body mass increased rapidly in mice fed with HFD. To determine whether celastrol reduced body mass in an obese asthmatic model, mice were weighed before and after celastrol intervention. At 14–15 week of age, higher body weight (∼40% higher) was observed in mice fed with a 45% fat diet (DIO+DMSO, DIO+OVA+DMSO, DIO+OVA+celastrol) compared to mice fed with a 10% fat (sham, OVA+DMSO) diet. Nevertheless, body weight significantly decreased in mice treated with celastrol [from (41.30 ± 1.958) g to (31.27 ± 1.611) g] compared to obese mice (40.98 ± 1.630 g) and obese asthmatic mice (40.97 ± 2.759 g) (**Figure 2**).

### Celastrol Ameliorates Airway Hyperresponsiveness (AHR) in Obese Asthmatic Mice

**FIGURE 3:**
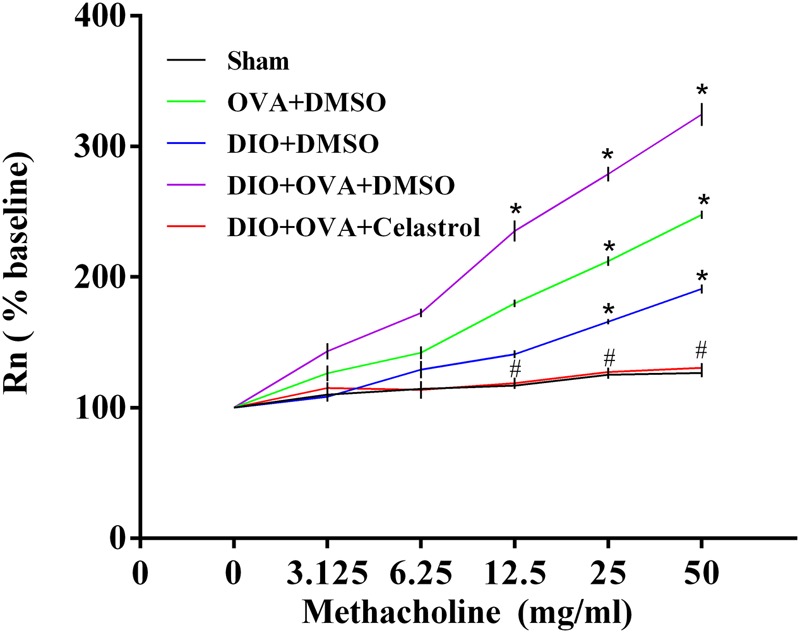
Celastrol ameliorates AHR of obese asthmatic mice. Airway resistance was determined by mouse ventilator and forced oscillation technique within 24 h after last challenge. Rn was measured at every concentration of methacholine. The data were expressed as mean ± SD (*n* = 6). ^∗^*P* < 0.05 compared with Sham group; ^#^*P* < 0.05 compared with DIO+OVA+DMSO group.

To explore the effect of celastrol on AHR in an obese asthmatic model, airway resistance (Rn) was detected. Briefly, Rn and MCh AHR was obviously higher in DIO+OVA+DMSO mice compared to Sham, OVA+DMSO and DIO+DMSO mice. However, these levels significantly decreased in mice treated with celastrol (**Table 1** and **Figure 3**).

**Table 1:** Celastrol reduces the airway resistence (Rn) of obese asthmatic mice.

Group	*n*	Rn (cmH_2_O/ml/s)
Sham	6	0.3503 ± 0.0032
OVA+DMSO	6	0.5463 ± 0.1226^∗∗^
DIO +DMSO	6	0.4483 ± 0.0010^∗^
DIO+OVA+DMSO	6	0.6550 ± 0.0972^∗∗^
DIO+OVA+Celastrol	6	0.4127 ± 0.0443^##^

*Airway resistence (Rn) was determined by Mouse ventilator and forced oscillation technique within 24 h after last challenge. The data were expressed as mean ± SD (*n* = 6). ^∗^*P <* 0.05; ^∗∗^*P <* 0.01 compared with Sham group; ^##^*P <* 0.01 compared with DIO+OVA+DMSO group.*

### Celastrol Ameliorates Airway Inflammation in Obese Asthmatic Mice

**FIGURE 4:**
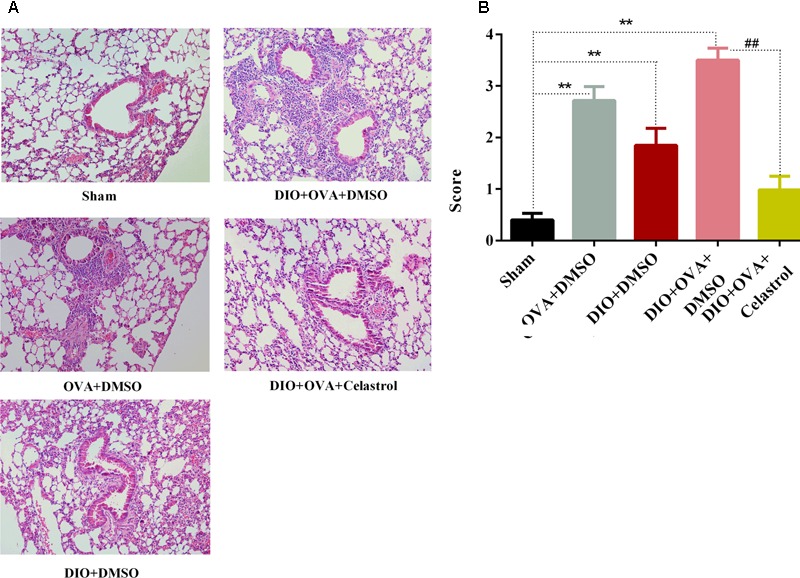
Celastrol ameliorates airway inflammation in obese asthmatic mice. **(A)** Lung tissues were stained with haematoxylin and eosin and analyzed under the light microscope (×200). **(B)** Semi-quantitative pathology scores among five groups. Data expressed as mean ± SD (*n* = 6). ^∗∗^*P* < 0.01 compared Sham group; ^##^*P* < 0.01 compared with DIO+OVA+DMSO group.

Significant higher inflammatory cells infiltration in the airways and small perivascular spaces of the lungs was observed in OVA+DMSO group, DIO+DMSO group, and DIO+OVA+DMSO group compared with Sham group and obese asthmatic mice treated with celastrol (**Figure 4**). In addition, significant infiltration of neutrophils, eosinophils and lymphocytes with marked thickening of airway wall and epithelial goblet cell metaplasia were detected in DIO+OVA+DMSO group compared with Sham group (**Figure 4**); while, reduced inflammation and airway wall thickening was observed in obese asthmatic mice treated with celastrol.

### Celastrol Decreases the Expression of IL-17A Protein in Lung Tissues

**FIGURE 5:**
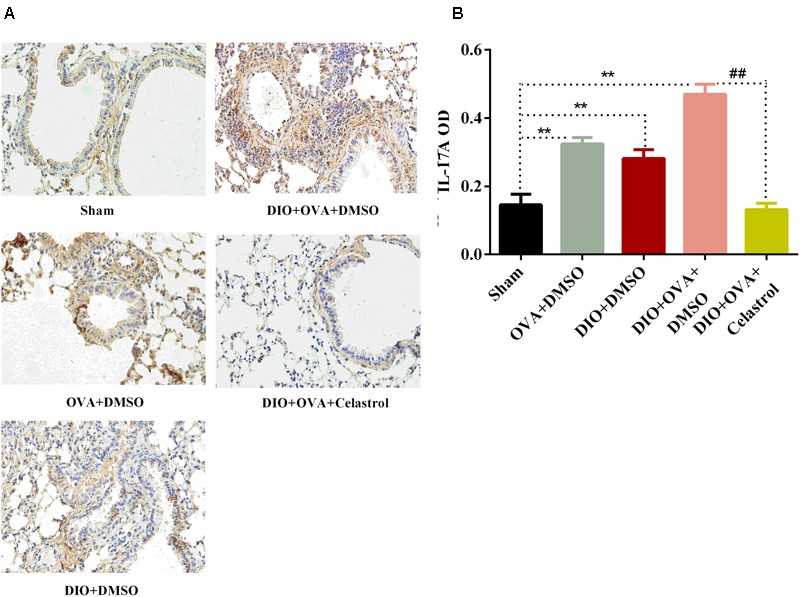
Celastrol decreases the expression of IL-17A protein in lung tissues. **(A)** IL-17A protein expression was detected by immunohistochemical imaging. **(B)** The protein of IL-17A was semi-quantified using Image-ProPlus software. Data were expressed as means ± SD. ^∗∗^*P* < 0.01 compared with Sham group; ^##^*P* < 0.01 compared with DIO+OVA+DMSO group.

IL-17A protein expression was assessed by immunohistochemical imaging and semi-quantified using Image-ProPlus software. The expression of IL-17A in lung tissues from DIO+OVA+DMSO group (0.4691 ± 0.0302), OVA+DMSO group (0.3238 ± 0.0190), and DIO+DMSO group (0.2811 ± 0.0262) significantly increased compared with Sham group (0.1452 ± 0.0310, *P* < 0.01). However, the expression of IL-17A protein in lung tissue significantly decreased in DIO+OVA+Celastrol group (0.1311 ± 0.0187, *P* < 0.01) (**Figure 5**).

### Celastrol Decreases the Frequency of Th17 Cells in the Spleen of Obese Asthmatic Mice

**FIGURE 6:**
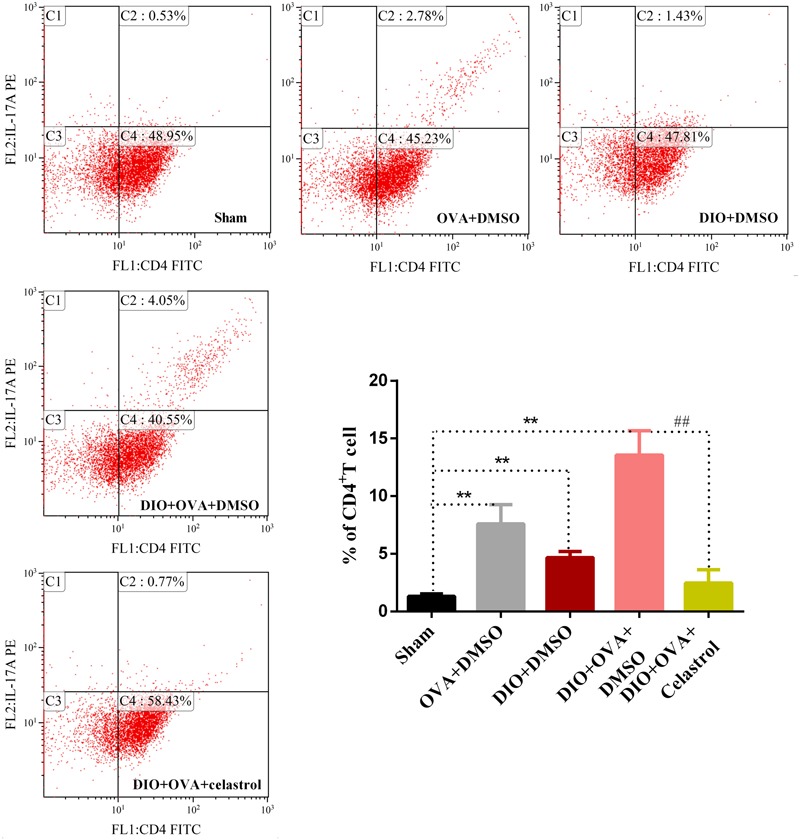
Celastrol treatment decreases the frequency of Th17 cell expansion. Proportion of Th17 cells in CD4^+^T cells was determined by flow cytometry. The data were expressed as mean ± SD (*n* = 6). ^∗∗^*P* < 0.01 compared with Sham group; ^##^*P* < 0.01 compared with DIO+OVA+DMSO group.

To explore the effect of celastrol on Th17 cell expansion, the proportion of Th17 cells in CD4^+^T cells from spleen was determined by flow cytometry. As shown in **Figure 6**, a significant increase of Th17 cells was found in the OVA+DMSO, DIO+DMSO, and DIO+OVA+DMSO group compared with the sham group (*P <* 0.01). Though, DIO+OVA+celastrol group revealed a significant reduction of Th17 cells compared with the Model (DIO+OVA+DMSO) group (*P <* 0.01).

### Celastrol Reduced the Serum Level of IL-17A in Obese Asthmatic Mice

**FIGURE 7:**
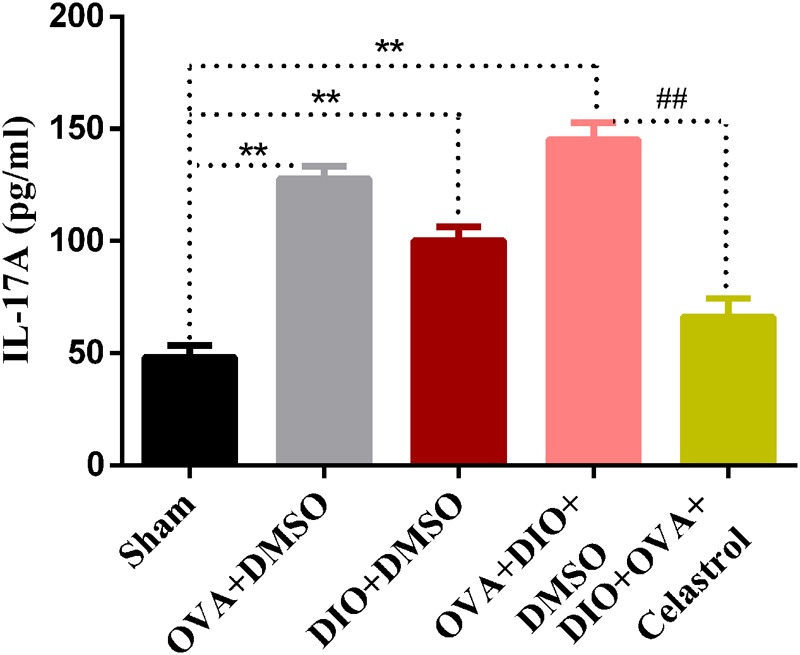
Celastrol reduces the production of IL-17A. IL-17A production were measured by ELISA assay. The data were expressed as mean ± SD (*n* = 6). ^∗∗^*P <* 0.01 compared with Sham group, ^##^*P <* 0.01 compared with DIO+OVA+DMSO group.

To further explore the effect of celastrol on Th17 cells, the production of IL-17A was measured by enzyme-linked immunosorbent assay. As indicated in **Figure 7**, OVA+DMSO and DIO+DMSO group (127.86 ± 5.56 pg/mL and 100.21 ± 6.12 pg/mL, *P* < 0.01) showed a higher level of IL-17A compared to sham group (48.25 ± 5.22 pg/mL). Interestingly, the highest levels of IL-17A were detected in the DIO+OVA+DMSO group (145.19 ± 7.61 pg/mL, *P* < 0.01). Contrary, IL-17A levels in serum from DIO+OVA+celastrol group were significantly reduced (66.24 ± 8.214 pg/mL, *P* < 0.01). We additionally measured serum OVA-specific IgE and IgG1, and found that there were no significant differences in OVA-specific IgE or IgG1 between OVA+DMSO group and DIO+OVA+DMSO group. Besides, either OVA-specific IgE or IgG1 level has no significant increase in DIO+DMSO group compared to Sham group (Supplementary Figure 2).

### Celastrol Affects the Expression of IL-17A mRNA in Lung of Obese Asthmatic Mice

**FIGURE 8:**
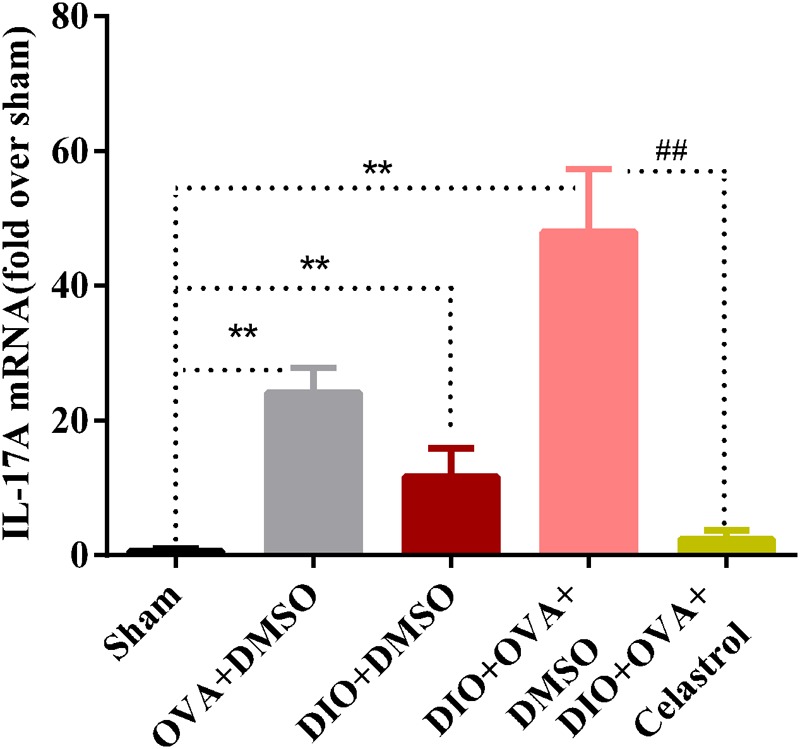
Celastrol reduces the mRNA expression of IL-17A in lung. Expression of IL-17A mRNA in lung was determined by quantitative real-time RT-PCR. The data were expressed as mean ± SD (*n* = 6). ^∗∗^*P* < 0.01 compared with Sham group; ^##^*P* < 0.01 compared with DIO+OVA+DMSO group.

To investigate the inhibition effects of celastrol on IL-17A, mRNA expression of IL-17A was examined. As shown in **Figure 8**, IL-17A mRNA expression was significantly higher in obese asthmatic mice (48.01 ± 9.31) compared to Sham, OVA+DMSO and DIO+DMSO (0.68 ± 0.30, 24.14 ± 3.66, and 11.70 ± 4.23, *P* < 0.01); while it was reduced in the DIO+OVA+celastrol (2.43 ± 1.24, *P* < 0.01).

### Correlation between AHR and Th17/CD4^+^T, and Th17 cytokines

**FIGURE 9:**
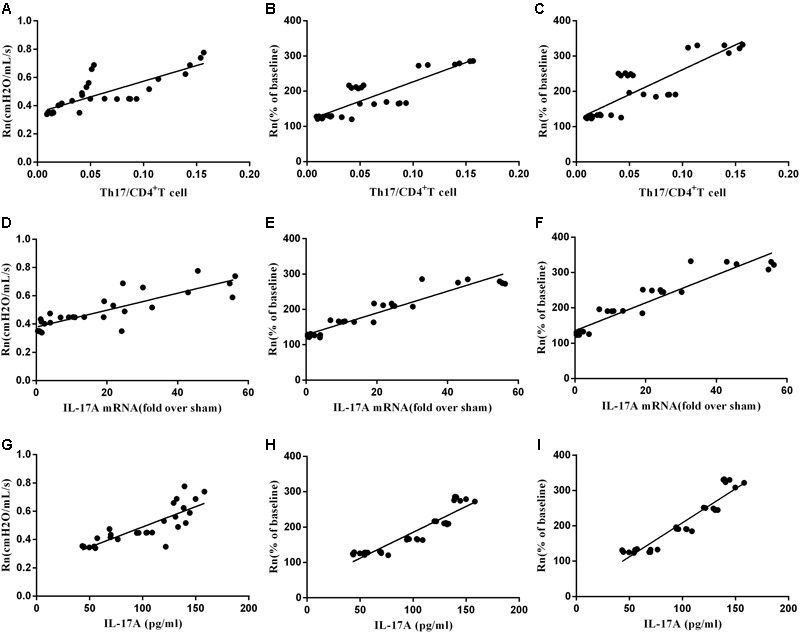
The correlation between AHR and Th17. **(A)** The correlation of baseline Rn andTh17/CD4^+^T cells (*n* = 30, *r* = 0.8401, *P <* 0.01). **(B)** The correlation of Mch (25 mg/mL) AHR and Th17/CD4^+^T cells (*n* = 30, *r* = 0.8189, *P <* 0.01). **(C)** The correlation of MCh (50 mg/mL) AHR and Th17/CD4^+^T cells (*n* = 30, *r* = 0.8363, *P <* 0.01). **(D)**The correlation of baseline Rn and IL17A mRNA (*n* = 30, *r* = 0.8408, *P <* 0.01). **(E)** The correlation of MCh (25 mg/mL) AHR and IL17A mRNA (*n* = 30, *r* = 0.8928, *P <* 0.01). **(F)** The correlation of MCh (50 mg/mL) AHR and IL17A mRNA (*n* = 30, *r* = 0.9137, *P <* 0.01). **(G)** The correlation of baseline Rn and IL-17A (*n* = 30, *r* = 0.8468, *P <* 0.01). **(H)** The correlation of MCh (25 mg/mL) AHR and IL-17A (*n* = 30, *r* = 0.9079, *P <* 0.01). **(I)** The correlation of MCh (50 mg/mL) AHR and IL-17A (*n* = 30, *r* = 0.9141, *P <* 0.01).

Finally, we determined the association between AHR and spleen Th17/CD4^+^T ratio, mRNA expression of IL-17A in lung, and serum IL-17A level of mice model. As seen in **Figure 9**, baseline Rn and MCh AHR (at 25 mg/mL and 50 mg/mL concentration) were both positively correlated to Th17 and Th17 cytokines in celastrol-treated or untreated mice, respectively.

## Discussion

Recently, an increase in the prevalence of childhood obesity and childhood asthma has been observed. This interaction has great potential to negatively affect the burden of asthma, in both individuals and populations. Asthma in obese children is usually more severe than in normal - weight individuals, and emerging data from clinical and translational studies suggest that obese patients are less likely to respond to controller therapies, particularly inhaled corticosteroids (Wenzel, 2012). Accordingly, finding the treatment for asthma in obesity is a matter of great importance. In the present study, we found that celastrol can reduce the body mass in obese asthmatic mice, and can ameliorate AHR and airway inflammation and reducing Th17 cells, expression of IL-17A mRNA and protein in lung and serum.

Recently, the role of celastrol in obesity has been reported by Ozcan and colleagues (Liu et al., 2015). In their study, C57BL/6 mice were orally treated with celastrol (10 mg/kg) for three weeks; their data suggested that celastrol has a robust effect on body weight reduction by increasing the sensitivity of leptin; in addition, celastrol has no toxic effect in mice. Moreover, it has been reported that AHR in obese asthma can be reduced by weight loss (Dixon et al., 2011; Chapman et al., 2014; Pakhale et al., 2015). In the present study, by observing the body mass changes in obese asthmatic mice treated with celastrol, we found that the body mass in mice decreased by 24% after 7 days of administration. This was significantly lower compared to both obese and obese-asthmatic mice, but still higher compared to sham group. We believe that the body mass would have reduced to the normal levels if the treatment with celastrol was extended. In addition, increasing evidence indicates that celastrol could be used as a therapeutic agent for allergy-induced asthma (Kim D.Y. et al., 2009; Kim Y. et al., 2009). Number of studies have shown that the obesity is usually accompanied by AHR (Arteaga-Solis et al., 2013; Kim et al., 2014). Moreover, obese asthma has shown to be accompanied by even more severe AHR (Kim et al., 2015), suggesting that AHR may be a common pathogenesis in obesity and asthma. Nonetheless, the optimal therapeutic approach has not been identified. In the present study, we established a DIO-OVA model, using mouse ventilator and forced oscillation technique to measure the airway resistance. The obtained results showed that airway resistance in obese asthmatic mice was the highest among all groups. Then MCh was used to make a challenge, and the airway resistance in both OVA and DIO mice increased faster compared to sham group as the concentration of MCh increased. The DIO-OVA mice increased the fastest, indicating that the severity of asthma worsens when accompanied by obesity. On the contrary, the airway resistance significantly decreased in celastrol-treated group, and it slowly increased when challenged by incremental concentration of MCh, which implied that celastrol ameliorates AHR in obese asthma.

Mathews et al. (2014) have found that mice fed with high-fat diets develop AHR, which in turn was correlated with Th17 cells and IL-17A levels. Kudo et al. (2012) have already demonstrated that the IL-17A produced by Th17 cells contributes to allergen-induced AHR by exerting direct effect on airway smooth muscle. Nevertheless, it remained unclear whether there was a correlation between Th17 cells and AHR in obese asthma. The present study furthermore revealed the proportion of Th17 cells in CD4^+^T lymphocytes from spleen. In addition, the expression of IL-17A mRNA and protein in lung and serum were significantly elevated in DIO-OVA, which expressed the highest airway resistance. The results indicated that Th17 and Th17 cytokines may play a critical role in AHR in obese asthma. The role of elevated Th17 cells and IL-17 levels in both asthmatic patients and OVA induced asthmatic mice have been previously demonstrated (Li et al., 2013; Kanellopoulou and Muljo, 2016). Likewise, it has previously been shown that inhibition of IL-17-related pathway could improve airway inflammation in asthma (Zhang et al., 2015). Th2 and Th17 cells can induce AHR, whereas Th17 cell-mediated airway inflammation and AHR are steroid resistant, indicating a potential role for Th17 cells in steroid-resistant asthma (McKinley et al., 2008); while increased levels of IL-17A lead to AHR (Barczyk et al., 2003). Interestingly, AHR appears to be dependent on IL-17A, since it does not develop in obese IL-17A^-/-^ mice (Kim et al., 2014). Th17 cell differentiation has also shown to be involved in the development of obesity (Endo et al., 2015), leptin induce RORγt expression and promotion of Th17 cell response (Yu et al., 2013). Th17 cells may be involved in chronic inflammation accompanying obesity (Sumarac-Dumanovic et al., 2009; Luczynski et al., 2015). Consequently, we believe that Th17 could be a promising therapeutic target for ameliorating AHR in obese asthma.

It has been reported that celastrol ameliorates experimental autoimmune encephalomyelitis (EAE) development by suppressing pathogenic Th17 responses (Wang et al., 2015). The effects of celastrol on rat adjuvant-induced arthritis (AA) through inhibition of Th17 cells have been demonstrated (Cascão et al., 2012; Astry et al., 2015). These findings indicated that celastrol may be an effective Th17 inhibitor. Consistent with these findings, our investigation revealed that treatment with celastrol markedly reduced the proportion of Th17 cells in CD4^+^T lymphocytes from spleen, decreased the expression of IL-17A mRNA and protein in lung, and decreased serum IL-17A levels in obese asthmatic mice. Then we determined the association beween AHR and spleen Th17/CD4^+^T ratio, lung mRNA expression of IL-17A and serum IL-17A level in mice model, and we found that either basline Rn or MCh AHR (at 25 mg/mL and 50 mg/mL concentration) was positively correlated to Th17 and Th17 cytokine in celastrol-treated or untreated mice. Taken together, our data strongly suggested that inhibition of Th17 could be an effective therapy for obese asthma. Of course, further preclinical and clinical researches are needed to address such potential.

## Conclusion

The current study proves that celastrol administration downregulates Th17 cells, decreases IL-17A production, and alleviates AHR in DIO-OVA mice. Therefore, our study provides a new insight into the treatment of obese asthma by celastrol.

## Author Contributions

ZZ and XL wrote the manuscript. WZ and ZZ contributed to designing the work. ZZ, RZ, and HZ performed the experiments. All authors read and approved the final version.

## Conflict of Interest Statement

The authors declare that the research was conducted in the absence of any commercial or financial relationships that could be construed as a potential conflict of interest.
