# Supplementary material for: Celastrol Alleviates Airway Hyperresponsiveness and Inhibits Th17 Responses in Obese Asthmatic Mice
Source: Front Pharmacol. 2018 Jan 31;9:49. doi: 10.3389/fphar.2018.00049 (PMC5797758; doi:10.3389/fphar.2018.00049)
Supplement: Supplementary file 1 [file Data_Sheet_1.pdf]

Supplementary Material

Celastrol Alleviates Airway Hyperresponsiveness and Inhibits Th17 Responses in Obese Asthmatic Mice

Zeyu Zeng*†, Xixi Lin*†, Rongying Zheng, Hui Zhang, Weixi Zhang*

*** Correspondence:** Weixi Zhang, zhangweixi112@163.com

†These authors contributed equally to this work

1 Supplementary Data

The post-hoc test utilized following ANOVA. Group 1 = Sham group, group 2 = OVA+DMSO group, group 3 = DIO+DMSO group, group 4 = DIO+OVA+DMSO group, group 5 = DIO+OVA+Celastrol group.

Multiple Comparisons

Dependent Variable: body mass (before celastrol treatment)
LSD

(I) group	(J) group	Mean Difference (I-J)	Std. Error	Sig.	95% Confidence Interval	
					Lower Bound	Upper Bound
1	2	-.76667	1.04837	.471	-2.9258	1.3925
	3	-13.25000*	1.04837	.000	-15.4092	-11.0908
	4	-13.46667*	1.04837	.000	-15.6258	-11.3075
	5	-13.41667*	1.04837	.000	-15.5758	-11.2575
2	1	.76667	1.04837	.471	-1.3925	2.9258
	3	-12.48333*	1.04837	.000	-14.6425	-10.3242
	4	-12.70000*	1.04837	.000	-14.8592	-10.5408
	5	-12.65000*	1.04837	.000	-14.8092	-10.4908
3	1	13.25000*	1.04837	.000	11.0908	15.4092
	2	12.48333*	1.04837	.000	10.3242	14.6425
	4	-.21667	1.04837	.838	-2.3758	1.9425
	5	-.16667	1.04837	.875	-2.3258	1.9925
4	1	13.46667*	1.04837	.000	11.3075	15.6258
	2	12.70000*	1.04837	.000	10.5408	14.8592
	3	.21667	1.04837	.838	-1.9425	2.3758
	5	.05000	1.04837	.962	-2.1092	2.2092
5	1	13.41667*	1.04837	.000	11.2575	15.5758
	2	12.65000*	1.04837	.000	10.4908	14.8092
	3	.16667	1.04837	.875	-1.9925	2.3258
	4	-.05000	1.04837	.962	-2.2092	2.1092

*. The mean difference is significant at the .05 level.

Multiple Comparisons

Dependent Variable: body mass (after celastrol treatment)
LSD

(I) group	(J) group	Mean Difference (I-J)	Std. Error	Sig.	95% Confidence Interval	
					Lower Bound	Upper Bound
1	2	-.10000	1.05303	.925	-2.2688	2.0688
	3	-13.43333*	1.05303	.000	-15.6021	-11.2646
	4	-13.41667*	1.05303	.000	-15.5854	-11.2479
	5	-3.71667*	1.05303	.002	-5.8854	-1.5479
2	1	.10000	1.05303	.925	-2.0688	2.2688
	3	-13.33333*	1.05303	.000	-15.5021	-11.1646
	4	-13.31667*	1.05303	.000	-15.4854	-11.1479
	5	-3.61667*	1.05303	.002	-5.7854	-1.4479
3	1	13.43333*	1.05303	.000	11.2646	15.6021
	2	13.33333*	1.05303	.000	11.1646	15.5021
	4	.01667	1.05303	.987	-2.1521	2.1854
	5	9.71667*	1.05303	.000	7.5479	11.8854
4	1	13.41667*	1.05303	.000	11.2479	15.5854
	2	13.31667*	1.05303	.000	11.1479	15.4854
	3	-.01667	1.05303	.987	-2.1854	2.1521
	5	9.70000*	1.05303	.000	7.5312	11.8688
5	1	3.71667*	1.05303	.002	1.5479	5.8854
	2	3.61667*	1.05303	.002	1.4479	5.7854
	3	-9.71667*	1.05303	.000	-11.8854	-7.5479
	4	-9.70000*	1.05303	.000	-11.8688	-7.5312

*. The mean difference is significant at the .05 level.

Multiple Comparisons

Dependent Variable: body mass reduction
LSD

(I) group	(J) group	Mean Difference (I-J)	Std. Error	Sig.	95% Confidence Interval	
					Lower Bound	Upper Bound
1	2	.66667	.75903	.388	-.8966	2.2299
	3	-.18333	.75903	.811	-1.7466	1.3799
	4	.05000	.75903	.948	-1.5133	1.6133
	5	9.70000*	.75903	.000	8.1367	11.2633
2	1	-.66667	.75903	.388	-2.2299	.8966
	3	-.85000	.75903	.273	-2.4133	.7133
	4	-.61667	.75903	.424	-2.1799	.9466
	5	9.03333*	.75903	.000	7.4701	10.5966
3	1	.18333	.75903	.811	-1.3799	1.7466
	2	.85000	.75903	.273	-.7133	2.4133
	4	.23333	.75903	.761	-1.3299	1.7966
	5	9.88333*	.75903	.000	8.3201	11.4466
4	1	-.05000	.75903	.948	-1.6133	1.5133
	2	.61667	.75903	.424	-.9466	2.1799
	3	-.23333	.75903	.761	-1.7966	1.3299
	5	9.65000*	.75903	.000	8.0867	11.2133
5	1	-9.70000*	.75903	.000	-11.2633	-8.1367
	2	-9.03333*	.75903	.000	-10.5966	-7.4701
	3	-9.88333*	.75903	.000	-11.4466	-8.3201
	4	-9.65000*	.75903	.000	-11.2133	-8.0867

*. The mean difference is significant at the .05 level.

Multiple Comparisons

Dependent Variable: Baseline Rn
LSD

(I) group	(J) group	Mean Difference (I-J)	Std. Error	Sig.	95% Confidence Interval	
					Lower Bound	Upper Bound
1	2	-.19598333*	.04199213	.000	-.2824677	-.1094989
	3	-.09803333*	.04199213	.028	-.1845177	-.0115489
	4	-.30481667*	.04199213	.000	-.3913011	-.2183323
	5	-.06236667	.04199213	.150	-.1488511	.0241177
2	1	.19598333*	.04199213	.000	.1094989	.2824677
	3	.09795000*	.04199213	.028	.0114656	.1844344
	4	-.10883333*	.04199213	.016	-.1953177	-.0223489
	5	.13361667*	.04199213	.004	.0471323	.2201011
3	1	.09803333*	.04199213	.028	.0115489	.1845177
	2	-.09795000*	.04199213	.028	-.1844344	-.0114656
	4	-.20678333*	.04199213	.000	-.2932677	-.1202989
	5	.03566667	.04199213	.404	-.0508177	.1221511
4	1	.30481667*	.04199213	.000	.2183323	.3913011
	2	.10883333*	.04199213	.016	.0223489	.1953177
	3	.20678333*	.04199213	.000	.1202989	.2932677
	5	.24245000*	.04199213	.000	.1559656	.3289344
5	1	.06236667	.04199213	.150	-.0241177	.1488511
	2	-.13361667*	.04199213	.004	-.2201011	-.0471323
	3	-.03566667	.04199213	.404	-.1221511	.0508177
	4	-.24245000*	.04199213	.000	-.3289344	-.1559656

*. The mean difference is significant at the .05 level.

Multiple Comparisons

Dependent Variable: Rn (% of baseline, 50 mg/mL)
LSD

(I) group	(J) group	Mean Difference (I-J)	Std. Error	Sig.	95% Confidence Interval	
					Lower Bound	Upper Bound
1	2	-121.20262*	2.892626	.000	-127.1600915	-115.2451419
	3	-64.365733*	2.892626	.000	-70.3232081	-58.4082585
	4	-198.04807*	2.892626	.000	-204.0055415	-192.0905919
	5	-4.1090333	2.892626	.168	-10.0665081	1.8484415
2	1	121.20262*	2.892626	.000	115.2451419	127.1600915
	3	56.836883*	2.892626	.000	50.8794085	62.7943581
	4	-76.845450*	2.892626	.000	-82.8029248	-70.8879752
	5	117.09358*	2.892626	.000	111.1361085	123.0510581
3	1	64.365733*	2.892626	.000	58.4082585	70.3232081
	2	-56.836883*	2.892626	.000	-62.7943581	-50.8794085
	4	-133.68233*	2.892626	.000	-139.6398081	-127.7248585
	5	60.256700*	2.892626	.000	54.2992252	66.2141748
4	1	198.04807*	2.892626	.000	192.0905919	204.0055415
	2	76.845450*	2.892626	.000	70.8879752	82.8029248
	3	133.68233*	2.892626	.000	127.7248585	139.6398081
	5	193.93903*	2.892626	.000	187.9815585	199.8965081
5	1	4.10903333	2.892626	.168	-1.8484415	10.0665081
	2	-117.09358*	2.892626	.000	-123.0510581	-111.1361085
	3	-60.256700*	2.892626	.000	-66.2141748	-54.2992252
	4	-193.93903*	2.892626	.000	-199.8965081	-187.9815585

*. The mean difference is significant at the .05 level.

Multiple Comparisons

Dependent Variable: Rn (% of baseline, 25 mg/mL)
LSD

(I) group	(J) group	Mean Difference (I-J)	Std. Error	Sig.	95% Confidence Interval	
					Lower Bound	Upper Bound
1	2	-87.039117*	2.156436	.000	-91.4803795	-82.5978539
	3	-40.638433*	2.156436	.000	-45.0796961	-36.1971705
	4	-153.71038*	2.156436	.000	-158.1516461	-149.2691205
	5	-2.1604667	2.156436	.326	-6.6017295	2.2807961
2	1	87.039117*	2.156436	.000	82.5978539	91.4803795
	3	46.400683*	2.156436	.000	41.9594205	50.8419461
	4	-66.671267*	2.156436	.000	-71.1125295	-62.2300039
	5	84.878650*	2.156436	.000	80.4373872	89.3199128
3	1	40.638433*	2.156436	.000	36.1971705	45.0796961
	2	-46.400683*	2.156436	.000	-50.8419461	-41.9594205
	4	-113.07195*	2.156436	.000	-117.5132128	-108.6306872
	5	38.477967*	2.156436	.000	34.0367039	42.9192295
4	1	153.71038*	2.156436	.000	149.2691205	158.1516461
	2	66.671267*	2.156436	.000	62.2300039	71.1125295
	3	113.07195*	2.156436	.000	108.6306872	117.5132128
	5	151.54992*	2.156436	.000	147.1086539	155.9911795
5	1	2.16046667	2.156436	.326	-2.2807961	6.6017295
	2	-84.878650*	2.156436	.000	-89.3199128	-80.4373872
	3	-38.477967*	2.156436	.000	-42.9192295	-34.0367039
	4	-151.54992*	2.156436	.000	-155.9911795	-147.1086539

*. The mean difference is significant at the .05 level.

Multiple Comparisons

Dependent Variable: Rn (% of baseline, 12.5 mg/mL)
LSD

(I) group	(J) group	Mean Difference (I-J)	Std. Error	Sig.	95% Confidence Interval	
					Lower Bound	Upper Bound
1	2	-62.904067*	2.724981	.000	-68.5162710	-57.2918623
	3	-23.993450*	2.724981	.000	-29.6056544	-18.3812456
	4	-118.17373*	2.724981	.000	-123.7859377	-112.5615290
	5	-1.8537500	2.724981	.503	-7.4659544	3.7584544
2	1	62.904067*	2.724981	.000	57.2918623	68.5162710
	3	38.910617*	2.724981	.000	33.2984123	44.5228210
	4	-55.269667*	2.724981	.000	-60.8818710	-49.6574623
	5	61.050317*	2.724981	.000	55.4381123	66.6625210
3	1	23.993450*	2.724981	.000	18.3812456	29.6056544
	2	-38.910617*	2.724981	.000	-44.5228210	-33.2984123
	4	-94.180283*	2.724981	.000	-99.7924877	-88.5680790
	5	22.139700*	2.724981	.000	16.5274956	27.7519044
4	1	118.17373*	2.724981	.000	112.5615290	123.7859377
	2	55.269667*	2.724981	.000	49.6574623	60.8818710
	3	94.180283*	2.724981	.000	88.5680790	99.7924877
	5	116.31998*	2.724981	.000	110.7077790	121.9321877
5	1	1.85375000	2.724981	.503	-3.7584544	7.4659544
	2	-61.050317*	2.724981	.000	-66.6625210	-55.4381123
	3	-22.139700*	2.724981	.000	-27.7519044	-16.5274956
	4	-116.31998*	2.724981	.000	-121.9321877	-110.7077790

*. The mean difference is significant at the .05 level.

Multiple Comparisons

Dependent Variable: Score of Histology Sections
LSD

(I) group	(J) group	Mean Difference (I-J)	Std. Error	Sig.	95% Confidence Interval	
					Lower Bound	Upper Bound
1	2	-2.3166667*	.14681810	.000	-2.6190442	-2.0142891
	3	-1.4500000*	.14681810	.000	-1.7523775	-1.1476225
	4	-3.1000000*	.14681810	.000	-3.4023775	-2.7976225
	5	-.58333333*	.14681810	.001	-.8857109	-.2809558
2	1	2.31666667*	.14681810	.000	2.0142891	2.6190442
	3	.86666667*	.14681810	.000	.5642891	1.1690442
	4	-.78333333*	.14681810	.000	-1.0857109	-.4809558
	5	1.73333333*	.14681810	.000	1.4309558	2.0357109
3	1	1.45000000*	.14681810	.000	1.1476225	1.7523775
	2	-.86666667*	.14681810	.000	-1.1690442	-.5642891
	4	-1.6500000*	.14681810	.000	-1.9523775	-1.3476225
	5	.86666667*	.14681810	.000	.5642891	1.1690442
4	1	3.10000000*	.14681810	.000	2.7976225	3.4023775
	2	.78333333*	.14681810	.000	.4809558	1.0857109
	3	1.65000000*	.14681810	.000	1.3476225	1.9523775
	5	2.51666667*	.14681810	.000	2.2142891	2.8190442
5	1	.58333333*	.14681810	.001	.2809558	.8857109
	2	-1.7333333*	.14681810	.000	-2.0357109	-1.4309558
	3	-.86666667*	.14681810	.000	-1.1690442	-.5642891
	4	-2.5166667*	.14681810	.000	-2.8190442	-2.2142891

*. The mean difference is significant at the .05 level.

Multiple Comparisons

Dependent Variable: IL-17A OD in Lung

LSD

(I) group	(J) group	Mean Difference (I-J)	Std. Error	Sig.	95% Confidence Interval Lower Bound	95% Confidence Interval Upper Bound
1	2	-.17865000*	.01476824	.000	-.2090658	-.1482342
	3	-.13596667*	.01476824	.000	-.1663824	-.1055509
	4	-.32395000*	.01476824	.000	-.3543658	-.2935342
	5	.01411667	.01476824	.348	-.0162991	.0445324
2	1	.17865000*	.01476824	.000	.1482342	.2090658
	3	.04268333*	.01476824	.008	.0122676	.0730991
	4	-.14530000*	.01476824	.000	-.1757158	-.1148842
	5	.19276667*	.01476824	.000	.1623509	.2231824
3	1	.13596667*	.01476824	.000	.1055509	.1663824
	2	-.04268333*	.01476824	.008	-.0730991	-.0122676
	4	-.18798333*	.01476824	.000	-.2183991	-.1575676
	5	.15008333*	.01476824	.000	.1196676	.1804991
4	1	.32395000*	.01476824	.000	.2935342	.3543658
	2	.14530000*	.01476824	.000	.1148842	.1757158
	3	.18798333*	.01476824	.000	.1575676	.2183991
	5	.33806667*	.01476824	.000	.3076509	.3684824
5	1	-.01411667	.01476824	.348	-.0445324	.0162991
	2	-.19276667*	.01476824	.000	-.2231824	-.1623509
	3	-.15008333*	.01476824	.000	-.1804991	-.1196676
	4	-.33806667*	.01476824	.000	-.3684824	-.3076509

*. The mean difference is significant at the .05 level.

Multiple Comparisons

Dependent Variable: Th17 (% of CD4+T Cell)
LSD

(I) group	(J) group	Mean Difference (I-J)	Std. Error	Sig.	95% Confidence Interval	
					Lower Bound	Upper Bound
1	2	-6.2776667*	.76973759	.000	-7.8629709	-4.6923624
	3	-3.3655000*	.76973759	.000	-4.9508042	-1.7801958
	4	-12.241333*	.76973759	.000	-13.8266376	-10.6560291
	5	-1.1688333	.76973759	.141	-2.7541376	.4164709
2	1	6.27766667*	.76973759	.000	4.6923624	7.8629709
	3	2.91216667*	.76973759	.001	1.3268624	4.4974709
	4	-5.9636667*	.76973759	.000	-7.5489709	-4.3783624
	5	5.10883333*	.76973759	.000	3.5235291	6.6941376
3	1	3.36550000*	.76973759	.000	1.7801958	4.9508042
	2	-2.9121667*	.76973759	.001	-4.4974709	-1.3268624
	4	-8.8758333*	.76973759	.000	-10.4611376	-7.2905291
	5	2.19666667*	.76973759	.009	.6113624	3.7819709
4	1	12.241333*	.76973759	.000	10.6560291	13.8266376
	2	5.96366667*	.76973759	.000	4.3783624	7.5489709
	3	8.87583333*	.76973759	.000	7.2905291	10.4611376
	5	11.072500*	.76973759	.000	9.4871958	12.6578042
5	1	1.16883333	.76973759	.141	-.4164709	2.7541376
	2	-5.1088333*	.76973759	.000	-6.6941376	-3.5235291
	3	-2.1966667*	.76973759	.009	-3.7819709	-.6113624
	4	-11.072500*	.76973759	.000	-12.6578042	-9.4871958

*. The mean difference is significant at the .05 level.

Multiple Comparisons

Dependent Variable: Serum IL-17A
LSD

(I) group	(J) group	Mean Difference (I-J)	Std. Error	Sig.	95% Confidence Interval	
					Lower Bound	Upper Bound
1	2	-79.610000*	3.837920	.000	-87.5143449	-71.7056551
	3	-51.965000*	3.837920	.000	-59.8693449	-44.0606551
	4	-96.938333*	3.837920	.000	-104.8426782	-89.0339885
	5	-17.991667*	3.837920	.000	-25.8960115	-10.0873218
2	1	79.610000*	3.837920	.000	71.7056551	87.5143449
	3	27.645000*	3.837920	.000	19.7406551	35.5493449
	4	-17.328333*	3.837920	.000	-25.2326782	-9.4239885
	5	61.618333*	3.837920	.000	53.7139885	69.5226782
3	1	51.965000*	3.837920	.000	44.0606551	59.8693449
	2	-27.645000*	3.837920	.000	-35.5493449	-19.7406551
	4	-44.973333*	3.837920	.000	-52.8776782	-37.0689885
	5	33.973333*	3.837920	.000	26.0689885	41.8776782
4	1	96.938333*	3.837920	.000	89.0339885	104.8426782
	2	17.328333*	3.837920	.000	9.4239885	25.2326782
	3	44.973333*	3.837920	.000	37.0689885	52.8776782
	5	78.946667*	3.837920	.000	71.0423218	86.8510115
5	1	17.991667*	3.837920	.000	10.0873218	25.8960115
	2	-61.618333*	3.837920	.000	-69.5226782	-53.7139885
	3	-33.973333*	3.837920	.000	-41.8776782	-26.0689885
	4	-78.946667*	3.837920	.000	-86.8510115	-71.0423218

*. The mean difference is significant at the .05 level.

Multiple Comparisons

Dependent Variable: Expression of IL-17A mRNA in Lung
LSD

(I) group	(J) group	Mean Difference (I-J)	Std. Error	Sig.	95% Confidence Interval	
					Lower Bound	Upper Bound
1	2	-23.464772*	2.823417	.000	-29.2797073	-17.6498364
	3	-11.022554*	2.823417	.001	-16.8374889	-5.2076181
	4	-47.329007*	2.823417	.000	-53.1439423	-41.5140714
	5	-1.7487387	2.823417	.541	-7.5636741	4.0661968
2	1	23.464772*	2.823417	.000	17.6498364	29.2797073
	3	12.442218*	2.823417	.000	6.6272829	18.2571538
	4	-23.864235*	2.823417	.000	-29.6791704	-18.0492996
	5	21.716033*	2.823417	.000	15.9010977	27.5309686
3	1	11.022554*	2.823417	.001	5.2076181	16.8374889
	2	-12.442218*	2.823417	.000	-18.2571538	-6.6272829
	4	-36.306453*	2.823417	.000	-42.1213888	-30.4915179
	5	9.27381483*	2.823417	.003	3.4588794	15.0887503
4	1	47.329007*	2.823417	.000	41.5140714	53.1439423
	2	23.864235*	2.823417	.000	18.0492996	29.6791704
	3	36.306453*	2.823417	.000	30.4915179	42.1213888
	5	45.580268*	2.823417	.000	39.7653327	51.3952036
5	1	1.74873867	2.823417	.541	-4.0661968	7.5636741
	2	-21.716033*	2.823417	.000	-27.5309686	-15.9010977
	3	-9.2738148*	2.823417	.003	-15.0887503	-3.4588794
	4	-45.580268*	2.823417	.000	-51.3952036	-39.7653327

*. The mean difference is significant at the .05 level.

Multiple Comparisons

Dependent Variable: OVA-IgE (U/mL)
LSD

(I) group	(J) group	Mean Difference (I-J)	Std. Error	Sig.	95% Confidence Interval Lower Bound	Upper Bound
1	2	-33.77333*	1.49456	.000	-36.8514	-30.6952
	3	-.56833	1.49456	.707	-3.6464	2.5098
	4	-34.89500*	1.49456	.000	-37.9731	-31.8169
	5	-14.97833*	1.49456	.000	-18.0564	-11.9002
2	1	33.77333*	1.49456	.000	30.6952	36.8514
	3	33.20500*	1.49456	.000	30.1269	36.2831
	4	-1.12167	1.49456	.460	-4.1998	1.9564
	5	18.79500*	1.49456	.000	15.7169	21.8731
3	1	.56833	1.49456	.707	-2.5098	3.6464
	2	-33.20500*	1.49456	.000	-36.2831	-30.1269
	4	-34.32667*	1.49456	.000	-37.4048	-31.2486
	5	-14.41000*	1.49456	.000	-17.4881	-11.3319
4	1	34.89500*	1.49456	.000	31.8169	37.9731
	2	1.12167	1.49456	.460	-1.9564	4.1998
	3	34.32667*	1.49456	.000	31.2486	37.4048
	5	19.91667*	1.49456	.000	16.8386	22.9948
5	1	14.97833*	1.49456	.000	11.9002	18.0564
	2	-18.79500*	1.49456	.000	-21.8731	-15.7169
	3	14.41000*	1.49456	.000	11.3319	17.4881
	4	-19.91667*	1.49456	.000	-22.9948	-16.8386

*. The mean difference is significant at the .05 level.

Multiple Comparisons

Dependent Variable: OVA-IgG1 (mg/mL)
LSD

(I) group	(J) group	Mean Difference (I-J)	Std. Error	Sig.	95% Confidence Interval	
					Lower Bound	Upper Bound
1	2	-10.02667*	1.12604	.000	-12.3458	-7.7075
	3	-.41333	1.12604	.717	-2.7325	1.9058
	4	-11.27667*	1.12604	.000	-13.5958	-8.9575
	5	-5.17500*	1.12604	.000	-7.4941	-2.8559
2	1	10.02667*	1.12604	.000	7.7075	12.3458
	3	9.61333*	1.12604	.000	7.2942	11.9325
	4	-1.25000	1.12604	.278	-3.5691	1.0691
	5	4.85167*	1.12604	.000	2.5325	7.1708
3	1	.41333	1.12604	.717	-1.9058	2.7325
	2	-9.61333*	1.12604	.000	-11.9325	-7.2942
	4	-10.86333*	1.12604	.000	-13.1825	-8.5442
	5	-4.76167*	1.12604	.000	-7.0808	-2.4425
4	1	11.27667*	1.12604	.000	8.9575	13.5958
	2	1.25000	1.12604	.278	-1.0691	3.5691
	3	10.86333*	1.12604	.000	8.5442	13.1825
	5	6.10167*	1.12604	.000	3.7825	8.4208
5	1	5.17500*	1.12604	.000	2.8559	7.4941
	2	-4.85167*	1.12604	.000	-7.1708	-2.5325
	3	4.76167*	1.12604	.000	2.4425	7.0808
	4	-6.10167*	1.12604	.000	-8.4208	-3.7825

*. The mean difference is significant at the .05 level.

2 Supplementary Figures and Tables

2.1 Supplementary Figures

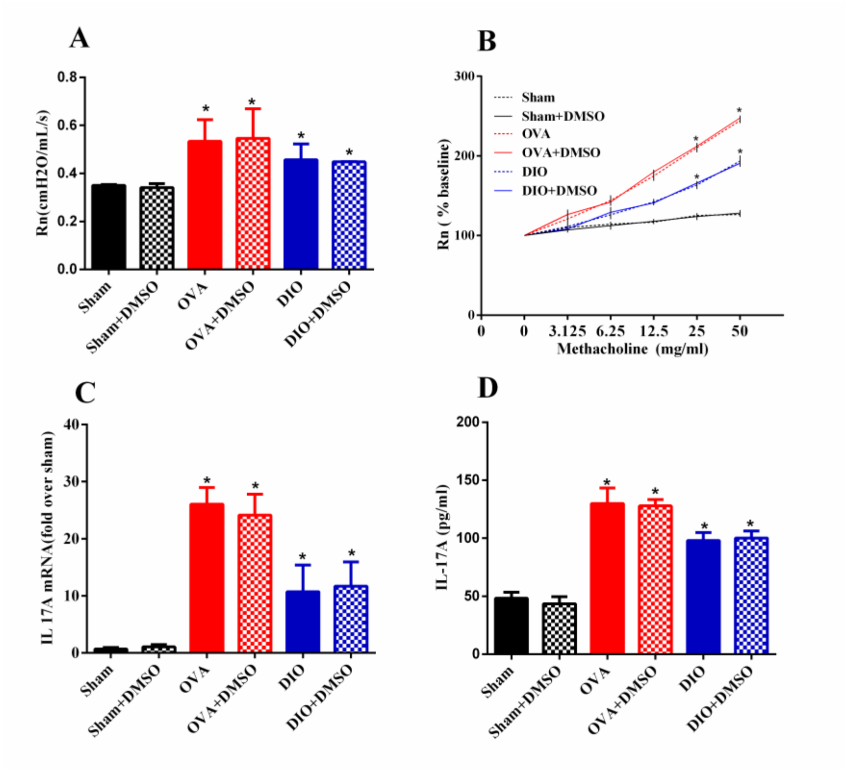

Supplementary Figure 1. Repetitive oral delivery of DMSO did not cause nonspecific inflammation. (A, B) Airway resistance was determined by mouse ventilator and forced oscillation technique within 24 hrs. after last challenge. Rn was measured at every concentration of methacholine. **(C)** Expression of IL-17A mRNA in the lung was determined by quantitative real-time RT-PCR. **(D)** IL-17A production was measured by ELISA assay. All the data were expressed as mean ± SD (n=6). $^{*}P < 0.05$ compared with Sham group.

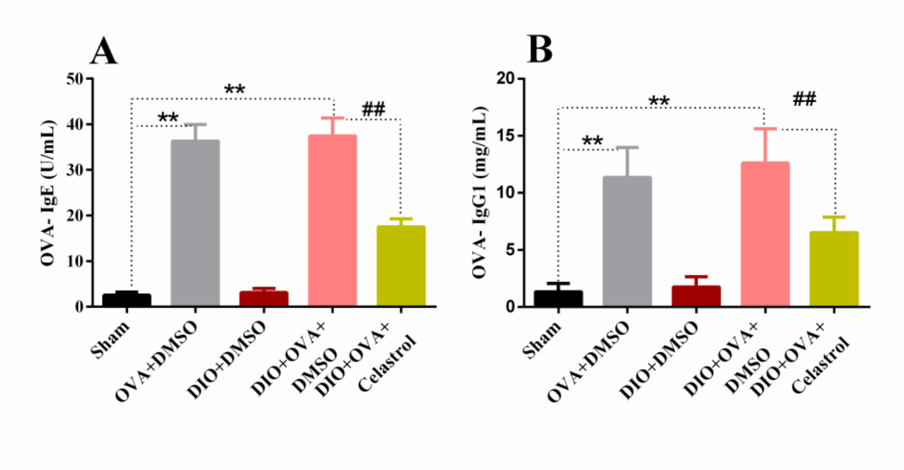

Supplementary Figure 2. Th2 specific antigens were not dominant in obese asthmatic mice. **(A)** Serum OVA-specific IgE and **(B)** IgG1 productions were measured by ELISA assay. The data were expressed as mean ± SD (n=6). $^{**}P$<0.01 compared with Sham group, $^{\#\#}P$<0.01 compared with DIO+OVA+DMSO group.
